# Complete Genome Sequence of Cellulase-Producing *Microbulbifer* sp. Strain GL-2, Isolated from Marine Fish Intestine

**DOI:** 10.1128/MRA.00746-20

**Published:** 2020-08-06

**Authors:** Yuta Sugimoto, Ken-ichiro Ohnishi, Satoru Suzuki

**Affiliations:** a Center for Marine Environmental Studies, Ehime University, Matsuyama, Japan; Georgia Institute of Technology

## Abstract

*Microbulbifer* sp. strain GL-2 was isolated from the intestine of a teleost, *Girella melanichthys.* Here, we report the complete genome sequence of this strain, which produces cellulase(s). Twelve cellulase candidate genes were found on the chromosome.

## ANNOUNCEMENT

The genus *Microbulbifer* has been reported from salty environments such as seawater (1), marine sediment (2), mangrove forest (3), marine algae (4), and sea cucumber gut (5). It is known that *Microbulbifer* isolates from mangrove sediment can degrade a variety of polysaccharides, including cellulose, and the whole genome has been reported (6). However, cellulase genes have not been identified in marine fish isolates.

*Microbulbifer* sp. strain GL-2, showing production of cellulase(s), was isolated from the gut of a smallscale blackfish (*Girella melanichthys*) (7). We used marine agar 2216 (Becton, Dickinson) with a 1:10 organic matter concentration for isolation. The medium contained 0.2% (wt/vol) AZCL-HE-cellulose (Megazyme) to detect cellulase activity. Plates were incubated at 25°C for 4 days. A single cellulase-positive colony was picked up. Classification of this strain was performed based on direct sequencing of the 16S rRNA gene with the use of a combination of primers (27f [5′-AGAGTTTGATCCTGGCTCAG-3′] and 907r [5′-CCGTCAATTCMTTTGAGTTT-3′], as well as 533f [5′-GTGCCAGCAGCCGCGGTAA-3′] and 1525r [5′-AGAAAGGAGGTGATCCAGCC-3′]). PCR products of the 16S rRNA gene were purified using a QIAquick PCR purification kit (Qiagen) and sequenced using a 3130 Genetic Analyzer (Applied Biosystems) with a BigDye Terminator v.3.1 cycle sequencing kit (Thermo Fisher Scientific). The sequence was confirmed by whole-genome sequencing. Here, we describe the complete genome sequence of this strain and the genes presumed to relate to cellulose-degrading enzymes.

Total DNA was extracted by using the Wizard genomic DNA purification kit (Promega) according to the manufacturer’s protocol, and a SMRTbell library was constructed. The genome sequencing of GL-2 was performed by shotgun sequencing using the PacBio RS II system (Pacific Biosciences) for the SMRTbell library. Quality control of reads was performed by filtering at a mean read score of 0.842 using RS_HGAP_Assembly.2 (SMRT Analysis v.2.3.0; Pacific Biosciences). The 103,587 sequence reads were pooled and *de novo* assembled using the same software (8). Default parameters were used for all software unless otherwise noted. A single contig was obtained, and the status of the contig was circular. The mean coverage was 144×. The complete genome of GL-2 was 4,950,731 bp, with an average G+C content of 49.6%. The Rapid Annotations using Subsystems Technology (RAST) server and SEED Viewer were used for gene annotation and an overview of the annotated genome, respectively (9, 10), and revealed 4,973 coding sequences (CDSs) and 70 RNA genes in this genome.

We queried the sequence for carbohydrate-active enzymes at the dbCAN2 database (11). Expected functions were confirmed in the NCBI database with BLASTp. A dbCAN2 search revealed candidate genes for cellulose-decomposing proteins, including glycosyl hydrolase family proteins (CDSs 2739, 2742, and 3301), glycoside hydrolase family proteins (CDSs 2739 and 2746), carbohydrate-binding proteins (CDSs 2744 and 3164), PKD domain-containing proteins (CDSs 4158 and 4160), an endo-1,4-β-glucanase (CDS 4156), a chitosanase (CDS 4155), and a PEP-CTERM sorting domain-containing protein (CDS 2745). Additionally, CDS 6 encoded a hypothetical protein with a cellulose-binding domain. This study provides information on new cellulase-producing bacteria and cellulose-degrading enzymes.

### Data availability.

The *Microbulbifer*. sp. strain GL-2 genome sequence has been deposited in DDBJ/EMBL/GenBank under accession number AP019807. Reads are available at the Sequence Read Archive (SRA) under accession number DRX180225 and BioSample number SAMD00177827 (SRA accession number DRA008880).
